# Modulation of the Gut Microbiome and Metabolomes by Fermentation Using a Probiotic Complex in a Dysbiosis-Associated Fecal Model

**DOI:** 10.4014/jmb.2506.06014

**Published:** 2025-11-26

**Authors:** Hayoung Kim, Hyeon Ji Jeon, Hye Min Jeong, Won Yeong Bang, Han Bin Lee, Kyu-Shik Lee, Jin Seok Moon, Hyeji Kwon, Jongkyun Lee, Jungwoo Yang, Young Hoon Jung

**Affiliations:** 1 Ildong Bioscience, Pyeongtaek-si 17957, Republic of Korea; 2 School of Food Science and Biotechnology, Food and Bio-industry Institute, Kyungpook National University, Daegu 41566, Republic of Korea; 3 Department of Pharmacology, College of Medicine, Dongguk University, Gyeongju 38066, Republic of Korea; 4 Department of Microbiology, College of Medicine, Dongguk University, Gyeongju 38066, Republic of Korea; 5 Cancer Genomic Research Institute, Seoul Song Do Colorectal Hospital, Seoul 38066, Republic of Korea; 6 Department of Surgery, Pelvic Floor Center, Seoul Song Do Colorectal Hospital, Seoul 38066, Republic of Korea

**Keywords:** gut dysbiosis, inflammatory bowel disease (IBD), probiotics, fecal fermentation, microbiome, metabolomics

## Abstract

Inflammatory bowel disease (IBD), affecting up to 0.5% of the global population, is frequently associated with gut microbiota dysbiosis and metabolic imbalances, which contribute to chronic constipation and abdominal discomfort. This study investigated the modulatory effects of an eight-strain probiotic complex comprising *Lactobacillus*, *Bifidobacterium*, and *Streptococcus* species on the gut microbiome and metabolome using an *in vitro* fecal fermentation model derived from a single IBD patient with dysbiosis. Metagenomic analysis demonstrated that increased abundance of beneficial bacteria, such as *Lacticaseibacillus rhamnosus*, while suppressing opportunistic pathogens, such as *Escherichia coli* and *Enterococcus faecium*. Metabolomic profiling further revealed significant alterations in metabolite levels that may help alleviate gut dysbiosis-related symptoms. These included increases in 3-hydroxybutyric acid, ascorbic acid, cadaverine, L-hydroxyproline, and N-acetylornithine and decreases in lysine and 3-aminoalanine. Given the single-donor design and the use of technical replicates, findings are presented as preliminary and descriptive rather than confirmatory. Collectively, these findings support the potential of probiotic fermentation to modulate microbial composition and metabolic output in a dysbiosis-associated context.

## Introduction

Gut dysbiosis refers to a chronic and recurrent imbalance of the intestinal microbial ecosystem, frequently associated with gastrointestinal disturbances such as imbalances in microbial metabolites, impaired intestinal barrier function, and dysregulation of the immune system. Its pathogenesis is multifactorial, involving diet and lifestyle factors, antibiotics and other medications, infections and inflammation, as well as host genetic predisposition [1]. Rather than representing a single disease, gut dysbiosis underlies a spectrum of gastrointestinal and systemic manifestations, characterized by phenotypic features that may overlap and evolve over time [2]. Dysbiosis not only disrupts local intestinal homeostasis but also exerts systemic effects through the gut-brain, gut-liver, and gut-immune axes, contributing to diverse clinical phenotypes [3].

Gut dysbiosis has emerged as a characteristic feature of gastrointestinal disorders such as inflammatory bowel disease (IBD) and irritable bowel syndrome (IBS) [4, 5]. In IBD, reduced microbial diversity and depletion of short-chain fatty acid (SCFA)–producing commensals such as *Faecalibacterium prausnitzii*, and *Bifidobacterium* spp. often occur with expansion of pro-inflammatory taxa including members of the Enterobacteriaceae [6, 7]. These alterations impair epithelial barrier integrity, increase intestinal permeability, and trigger inappropriate immune activation, ultimately driving chronic intestinal inflammation [8]. Similar dysbiotic signatures have been also described in IBS by reduction in beneficial genera such as *Lactobacillus* and *Bifidobacterium*, accompanied by an expansion of potentially pathogenic taxa such as *Firmicutes* and Proteobacteria [9, 10].

Interventions to modulate dysbiosis include dietary modifications, probiotics, prebiotics, synbiotics, and fecal microbiota transplantation [11]. While these interventions have demonstrated potential in restoring microbial balance, enhancing barrier integrity, and alleviating gastrointestinal symptoms, clinical outcomes remain variable across individuals [12]. Moreover, the efficacy and safety of long-term probiotic or synbiotic supplementation are not fully established, underscoring the need for mechanism-informed strategies [13]. These limitations highlight the need for well-defined, mechanism-driven strategies that provide durable and safe modulation of gut microbiota.

While many studies have examined single-strain probiotics, their effects are strain-specific and often modest or transient, making it difficult to achieve long-term stabilization of the gut microbial ecosystem [14]. Host-related factors such as genetics, diet, and baseline microbiota composition further influence efficacy, leading to considerable inter-individual variability in clinical response. In contrast, multi-strain probiotics can target diverse metabolic and immunological pathways, allowing for more comprehensive modulation of the gut environment [15]. Complementary interactions among strains may generate synergistic effects that are rarely achieved with single strains, thereby strengthening barrier function and restoring immune balance [16, 17]. Accordingly, multi-strain probiotics have emerged as a more promising strategy for restoring microbial balance and improving clinical outcomes.

The gut microbiome comprises the microorganisms that live in the intestines, the surrounding ecosystem, and the interactions between the two [18]. Defining a healthy gut microbiome is challenging, as there are no specific criteria, and different bacterial species share similar functions in maintaining gut health [19]. However, the use of probiotics is a well-established approach to maintaining gut microbiome health [20, 21]. Many studies have investigated the effects of fermentation using probiotics in fecal samples from patients with gut dysbiosis [22]. However, the precise mechanisms by which such interventions exert therapeutic effects remain incompletely understood. Moreover, given the multifactorial pathophysiology of gut dysbiosis—including altered SCFA metabolism, mucosal barrier dysfunction, and visceral hypersensitivity—the use of a multi-strain probiotic complex may offer synergistic benefits.

Here, we evaluated a representative multi-strain probiotic complex—consisting of *Bifidobacterium*, *Lactobacillus*, and *Streptococcus* species—in an *in vitro* fecal fermentation model derived from a single IBD patient with dysbiosis. We assessed SCFA production, microbiome composition, and metabolite profiles relative to unfermented controls.

## Materials and Methods

### Study Subjects and Fecal Sample Collection

This study was conducted using fecal samples collected from an adult IBD patient exhibiting gut dysbiosis. The samples were collected using a dedicated collection kit (AccuStool Collection Kit, AccuGene, Republic of Korea) and immediately stored at −80°C. The collection of fecal samples was approved by the Institutional Review Board of Seoul Songdo Hospital (approval number: 2021-004). For the *in vitro* colonic fermentation, a single fecal sample obtained from one IBD patient was used. The clinicopathological characteristics of the fecal donor were as follows: male, aged 25, clinically diagnosed with IBD, with an anal fistula, and no antibiotic or probiotic use within 8 weeks prior to sample collection. All analyses were performed using technical replicates prepared from this single donor sample. In addition, clinical disease severity in IBD patients, particularly those with Crohn’s disease was assessed using the Montreal classification, and endoscopic disease severity was evaluated using the SES-CD (Simple Endoscopic Score for Crohn Disease). Crohn’s disease is a subtype of IBD characterized by chronic inflammation of the gastrointestinal tract, which can lead to abdominal pain, severe diarrhea, fatigue, weight loss, and malnutrition.

### *In Vitro* Colonic Fermentation

For *in vitro* colonic fermentation, fecal samples were respectively used as inocula for single bacterial cultures or bacterial mixed cultures. Phosphate-buffered saline (PBS; pH 7.4) was used to dilute the feces (ratio 1:10; w/v), and the mixture was homogenized using a Bag Mixer 400 SW (Interscience, France) for 10 min. A fermentation basal medium consisting of 0.01 g/l of calcium chloride hexahydrate (CaCl_2_·6H_2_O), 0.04 g/l of potassium phosphate monobasic (KH_2_PO_4_), 0.01 g/l of magnesium sulfate heptahydrate (MgSO_4_·7H_2_O), 2 g/l of sodium bicarbonate (NaHCO_3_), 0.1 g/l of sodium chloride (NaCl), 0.5 g/l of L-cysteine hydrochloride, 2 ml/l of Tween 80, 2 g/l of yeast extract, 0.05 g/l of hemin, 10 μl/l of vitamin K, 0.5 g/l of bile salts, and 2 g/l of peptone water was prepared. It was adjusted to a pH of 7.0, and then 4 ml/l of 0.025% (w/v) resazurin solution was added and the medium was autoclaved.

**Fig. 1:**
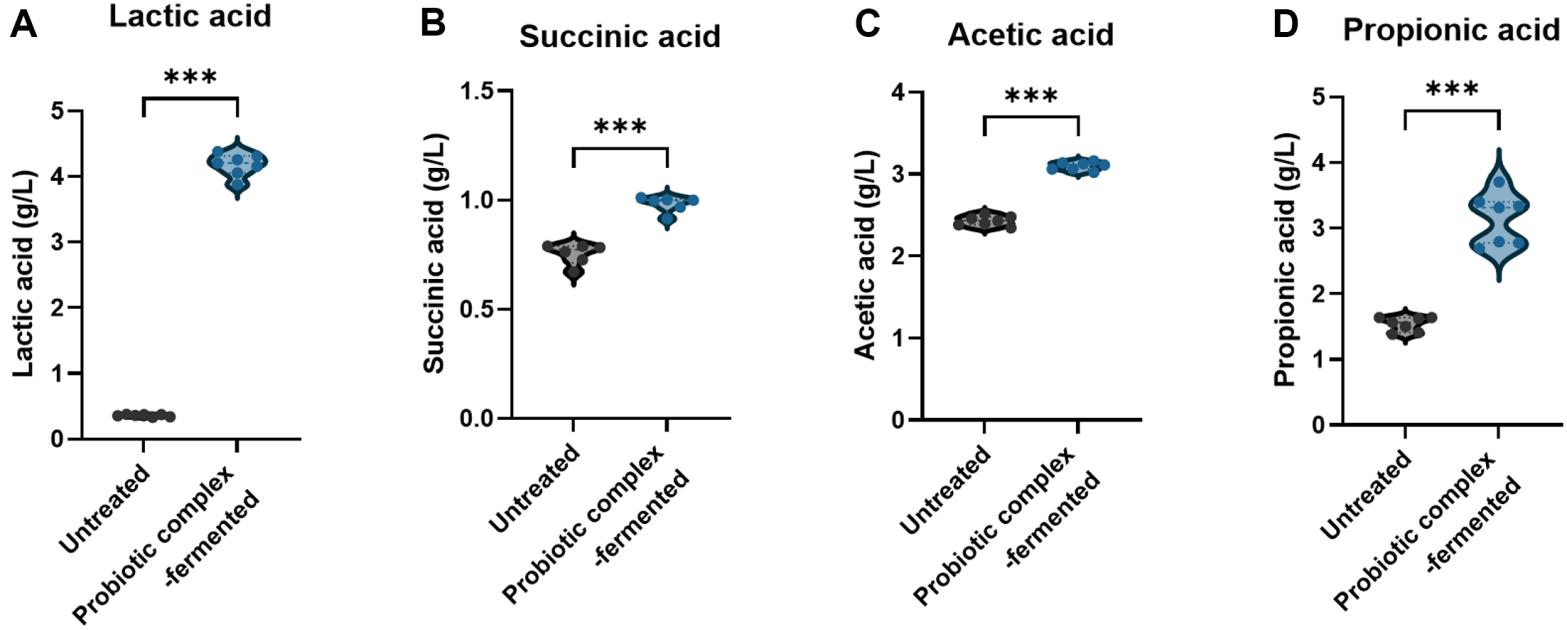
SCFA production in untreated and probiotic complex-fermented IBD fecal samples. (**A**) Lactic acid and (**B**) succinic acid both as SCFA precursors, (**C**) acetic acid and (**D**) propionic acid were included. Statistical significance is indicated as follow: *p* < 0.05 (*), *p* < 0.01(**), and *p* < 0.001 (***)

For the probiotic treatment, 5 ml of prepared fecal slurry was mixed with 45 ml of basal medium to create the colonic medium. A probiotic mixture comprising eight strains—*Lacticaseibacillus rhamnosus* IDCC 3201, *Lactiplantibacillus plantarum* IDCC 3501, *Lacticaseibacillus casei* IDCC 3451, *Bifidobacterium animalis* subsp. *lactis* IDCC 4301, *Bifidobacterium breve* IDCC 4401, *Lactobacillus helveticus* IDCC 3801, *Limosilactobacillus reuteri* IDCC 3701, and *Streptococcus thermophilus* IDCC 2201—all provided by Ildong Bioscience (Republic of Korea), was inoculated into the colon medium at a concentration of 1 × 10^9^ CFU/ml, and the culture was incubated at 37°C for 24 h. Single probiotics, used as probiotic mixture, were also prepared for IBD fecal fermentation at a concentration of 1 × 10^9^ CFU/ml. After incubation, the cultures were centrifuged at 8,000 rpm for 10 min. The bacterial cell pellet was used for metagenomic analysis, while the supernatant was used for the analyses of SCFAs and other metabolites (Fig. 1).

### Quantification of Short-Chain Fatty Acids

The colonic fermentation supernatant was concentrated using a vacuum concentrator (Eppendorf Concentrator Plus, Germany). Then, the sample was filtered through a 0.45 μm syringe filter and analyzed via high-performance liquid chromatography (HPLC; Agilent 1260, Agilent Technologies, USA), with an Aminex HPX-87H column (300 mm × 7.8 mm, 9-μm particle size; Bio-Rad Laboratories, USA) used for the separation and quantification of SCFAs. The mobile phase, consisting of 0.005 N sulfuric acid, was run at a flow rate of 0.6 ml/min. The column was maintained at 60°C, and 10 μl of each sample was injected. Peaks were detected using a UV detector 210 nm.

### Metagenomic Analysis

Total microbial metagenomic DNA (mDNA) was extracted from the bacterial cells using the QIAamp PowerFecal Pro DNA Kit (QIAGEN Co., Germany) according to the manufacturer’s instructions. Full-length 16S rRNA genes were amplified using universal primers 27F (5'-AGRGTTYGATYMTGGCTCAG-3') and 1492R (5'-RGYTACCTTGTTACGACTT-3'), each tagged with sample-specific PacBio barcodes. The amplified 16S rRNA products were prepared as libraries using the SMRTbell Express Template Prep Kit 2.0 (Pacific Biosciences, USA), following the manufacturer’s instructions, and sequencing was performed on a PacBio Sequel II platform. Circular consensus sequence (CCS) HiFi reads were generated and demultiplexing was conducted using PacBio SMRT Link software. The sequenced HiFi reads were preprocessed using QIIME 2 (v.2022.2) and the *DADA2* R package (v.1.18). Reads ranging from 1,000–1,600 bp in length were selected using the "qiime dada2 denoise-ccs" command, and barcode and primer sequences were removed. Subsequently, amplicon sequence variant (ASV) tables were generated with *DADA2*, and taxonomy classification at genus and species levels was performed using the SILVA rRNA database (v.138.99). A phylogenetic analysis was conducted in QIIME 2, and the resulting phylogenetic tree was used in subsequent downstream analyses.

Alpha diversity was assessed by calculating the Chao1, Shannon, and Simpson indices within the QIIME 2 platform. Alpha and beta diversity analyses were conducted at the ASV level. Given the limited sample size (n =3 per group), we report median and interquartile ranges without inferential statistical testing to avoid overinterpretation. For beta diversity, the *Phyloseq* R package was used to perform a principal coordinates analysis (PCoA) based on unweighted UniFrac and weighted UniFrac distances. The relative abundances of microbial taxa were visualized at the phylum, genus and species levels. Furthermore, linear discriminant analysis effect size (LEfSe) analysis was employed to identify bacterial species with significantly different abundances between groups, combining non-parametric statistical testing (Kruskal–Wallis test) with effect size estimation using linear discriminant analysis (LDA). Taxa with an adjusted *p* < 0.05 and an LDA score ≥ 2 were considered significant.

### Metabolite Extraction

Metabolites were extracted from the colonic fermentation sample supernatants for metabolomic analyses. For each 750 μl of sample, 2.25 ml of ice-cold methanol was added, and the mixture was vortexed for 1 min on ice. After centrifugation at 10,000 rpm for 10 min at 4°C, the supernatant was collected and filtered through a 0.2 μm PVDF syringe filter. One hundred microliter of the filtered samples was then transferred into a 1.5 ml microtube and dried using in a vacuum concentrator (Eppendorf Concentrator Plus, Germany).

### Gas Chromatography-Mass Spectrometry Analysis

The dried samples were derivatized by adding 50 μl of 20 mg/ml methoxyamine hydrochloride solution in pyridine (Sigma, USA) and incubating for 90 min at 30°C. Subsequently, 100 μl of N, O-bis(trimethylsilyl) trifluoroacetamide (BSTFA; Sigma) was added, and the samples were heated for 30 min at 60°C. An alkane standards mixture and fluoranthene were used as retention index markers and an internal standard, respectively.

A gas chromatography-mass spectrometry analysis was conducted using a Chromatec-Crystal 5000 gas chromatograph (Chromatec, Mari El, Russia) coupled to a Chromatec-Crystal 5000 mass spectrometer (Chromatec) equipped with a J&W VF-5ms GC column (60 mm × 0.25 mm, 0.25-μm film thickness; Agilent Technologies). Derivatized samples were injected at 280°C using a split ratio of 1:10, and metabolites were separated using a 5 ml/min helium flow employing an oven program consisting of 2 min at 50°C, ramping up to 180°C at 5°C/min, 8 min at 180°C, ramping up to 210°C at 2.5°C/min, ramping up to 320°C at 5°C/min, and then 10 min at 320°C. Mass spectra were acquired in the scan range of 35–650 m/z at 0.2 spectra per sec in standard mode with an ion source temperature of 280°C. Spectra were processed using Thermo Xcalibur software with automated peak detection, and metabolites were identified by matching mass spectra and retention indices using the NIST Mass spectral library (v.2.4, USA) and MS-DIAL (http://prime.psc.riken.jp/compms/msdial/main.html). Relative metabolite intensities were normalized to the sum of identified peaks.

### Statistical Analysis

Metabolite data were evaluated using paired *t*-tests performed in GraphPad Prism 10 (USA). Differences were considered significant when *p*-values < 0.05 (*) or < 0.01 (**). Multivariate analyses and heatmap generation were performed using MetaboAnalyst 5.0 (https://www.metaboanalyst.ca/).

## Results

### SCFA Production in Gut Dysbiosis-associated Fecal Samples after Fermentation with a Probiotic Complex

Since SCFAs are primary microbial metabolites produced by gut microbiota and their levels can be compared to estimate gut health [23], the production of SCFAs, including succinic acid, lactic acid, acetic acid, and propionic acid, was measured in untreated and probiotic-fermented fecal samples (Fig. 1). Fermentation with the probiotic complex resulted in the increased production of all SCFAs. Specifically, lactic acid rose from 0.36 ± 0.02 g/l to 4.18 ± 0.17 g/l. Succinic acid, acetic acid, and propionic acid also increased after fermentation, from 0.71 ± 0.12 g/l, 2.43 ± 0.07 g/l, and 1.53 ± 0.11 g/l to 1.13 ± 0.81 g/l, 2.87 ± 1.07 g/l, and 2.73 ± 0.96 g/l, respectively.

The production of SCFAs is influenced by microbial composition [24], highlighting the importance of investigating changes in the fecal microbiome after probiotic fermentation. Additionally, correlations between SCFA concentrations and the predominant microbial species present in fecal samples might help clarify the mechanisms underlying the role of probiotics in mediating metabolic modulation.

### Metagenomic Profiles of Gut Dysbiosis-Associated Fecal Samples after Fermentation with a Probiotic Complex

**Fig. 2:**
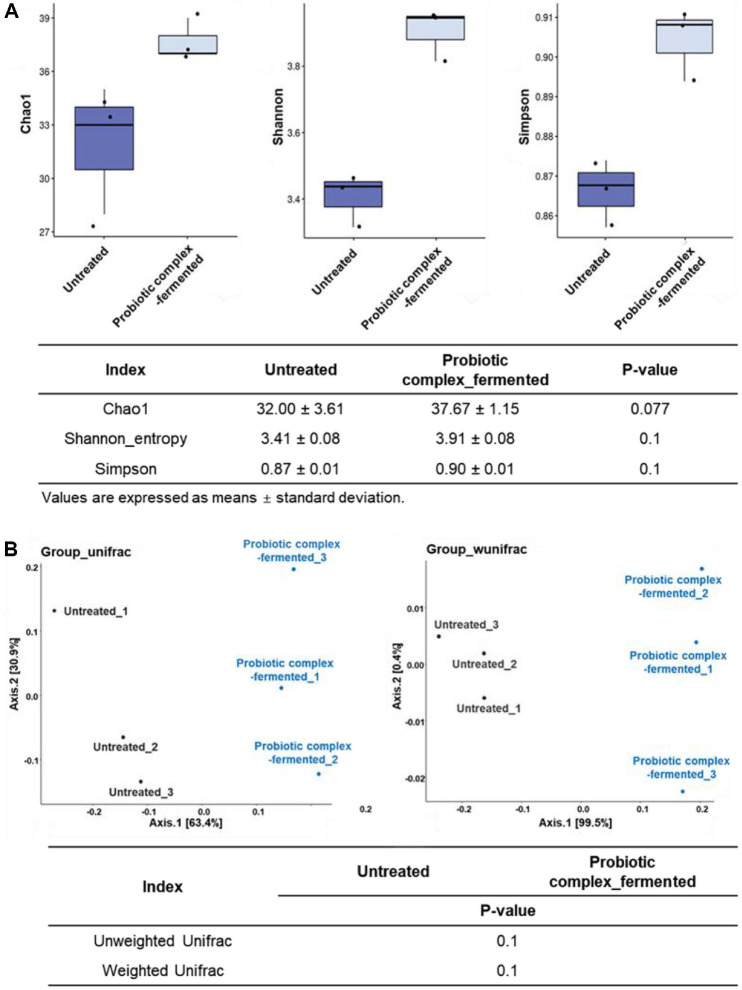
Microbial community diversity in probiotic complex-fermented IBD fecal samples: (**A**) alpha diversity (Chao1, Shannon, and Simpson indices) and (**B**) beta diversity (PCoA biplots based on the Group_unifrac and Group_wunifrac distances).

Metagenomic sequencing was performed to examine changes in microbial composition and abundance after the fermentation of gut dysbiosis-associated fecal samples with an eight-strain probiotic complex. Alpha and beta diversity analyses revealed the changes to microbial community compositions and abundances after fermentation, but there was no statistical significance using Kruskal–Wallis test (*p* > 0.05) (Fig. 2). The increases in alpha diversity suggest that fermentation enhanced the microbiome diversity of gut dysbiosis-associated patients’ feces (Fig. 2A). Specifically, probiotic complex-fermented fecal samples exhibited higher Chao1 and Shannon indices, indicating greater taxon richness and evenness in the fecal microbiomes, respectively. These results suggest that fermentation with the probiotic complex contributed to a more diverse and balanced microbial community. By contrast, Simpson’s index, which measures species dominance, showed that fermentation reduced the dominance of a few species, promoting a more balanced microbial composition in the gut dysbiosis-associated fecal samples.

The beta diversity analysis also revealed the differences between the microbiomes of the untreated and probiotic complex-fermented fecal samples (Fig. 2B). A PCoA based on unweighted UniFrac distances, which measure phylogenetic differences between groups, indicated that gut dysbiosis-associated fecal samples exhibited distinct microbial communities following fermentation using the probiotic complex. Higher weighted UniFac distances indicate greater phylogenetic and abundance differences between microbial communities. Thus, the distinct separation of untreated and fermented fecal samples seen in the PCoA biplot based on this measure reveals significant alterations in both the composition and abundance of fecal microbiota.

Although the statistical significance of metagenomic data was not available due to the small number of samples (three replicates), we do not interpret *p*-values as evidence of confirmatory significance. Therefore, specific microbial composition and LDA analyses were necessary to further explore which microorganisms contribute to the differences between the two groups.

**Fig. 3:**
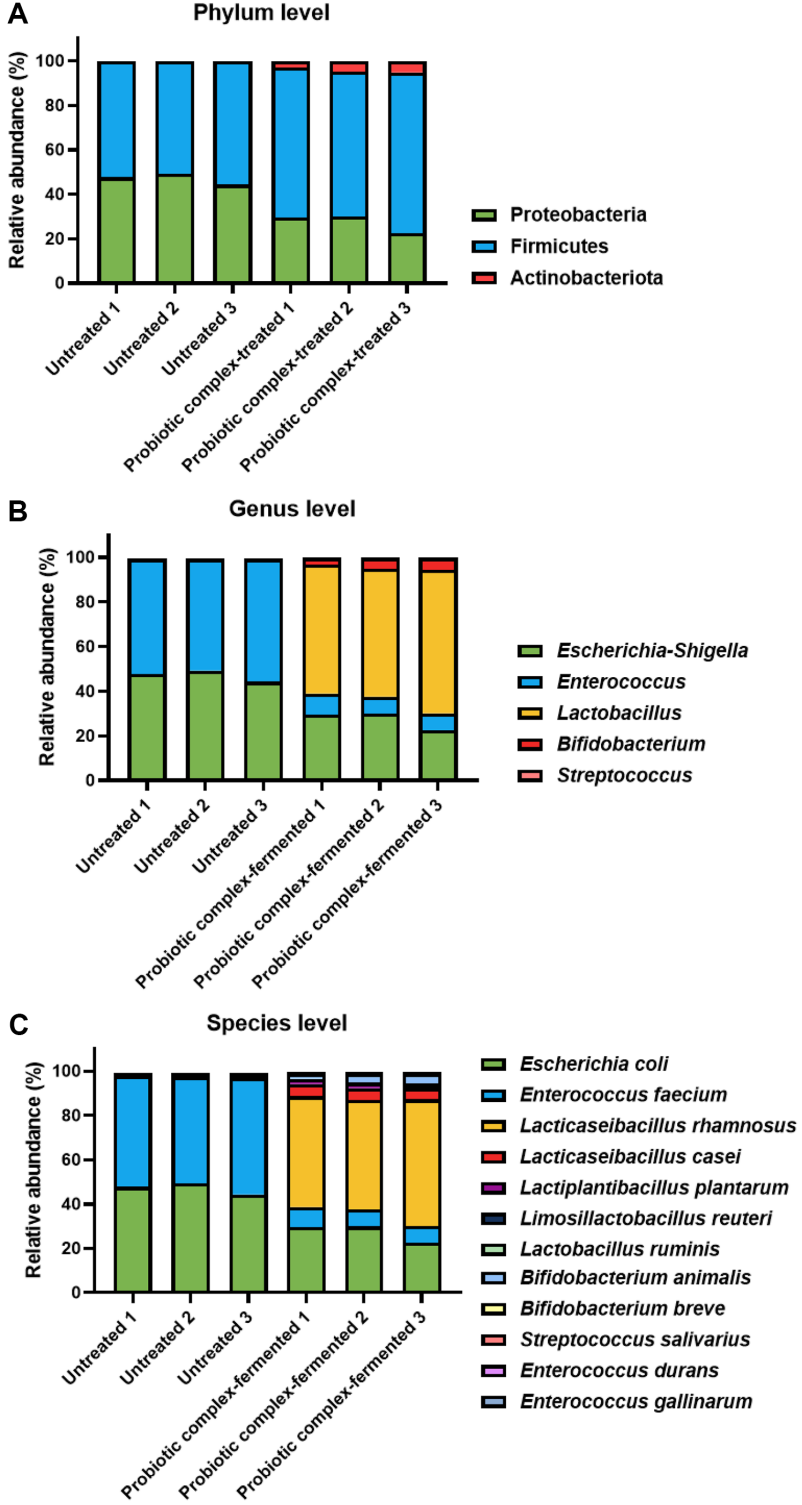
Taxonomic composition of probiotic complex-fermented IBD fecal samples: the relative abundances (%) of microbial taxa at the (**A**) phylum, (**B**) genus, and (**C**) species levels.

Taxonomic analysis of the gut dysbiosis-associated fecal microbiome was performed at the phylum, genus, and species levels to elucidate the changes in microbial community composition induced by fermentation with the probiotic complex (Fig. 3). A total of three phyla were identified in untreated fecal samples, with the overall fecal microbiota of gut dysbiosis-associated patients dominated by taxa from *Firmicutes* and Proteobacteria and a small contribution from *Actinobacteria*, regardless of fermentation. However, the relative proportions of each phylum changed following the probiotic complex fermentation (Fig. 3A). The relative abundances of *Firmicutes* and *Actinobacteria* increased from 52.75 ± 2.51% to 68.31 ± 3.60% and from 0.17 ± 0.02% to 4.38 ± 1.25%, respectively, after fermentation, while that of Proteobacteria decreased from 47.08 ± 2.51% to 27.31 ± 4.17%.

At the genus level, 13 genera were detected, eight of which—*Anaerostipes*, *Blautia*, *Clostridium*, *Dorea*, *Erysipelotrichaceae*, *Eubacterium*, *Subdoligranulum*, and *Ruminococcus* spp.—displayed non-significant changes in relative abundance after probiotic complex fermentation (grouped as “Other” in Fig. 3B). Of the other five, *Enterococcus* and *Escherichia*-*Shigella* decreased, while *Lactobacillus*, *Bifidobacterium*, and *Streptococcus* increased due to the fermentation treatment (Fig. 3B). The genus with the greatest decrease in relative abundance was *Enterococcus*, which was the dominant genus in untreated fecal samples. It represented 52.16 ± 2.46% of the microbial community in untreated samples, but only 8.23 ± 0.98% after fermentation. The relative abundance of *Escherichia*-*Shigella* decreased from 47.08 ± 2.51% in untreated samples to 27.31 ± 4.17% in probiotic complex-fermented samples. Conversely, the genus with the highest increase in relative abundance during fermentation was *Lactobacillus*, increasing from 0.19 ± 0.02% to 59.77 ± 3.93% and becoming the dominant taxon. The relative abundance of *Bifidobacterium* and *Streptococcus* also increased, from 0.17 ± 0.02% to 4.38 ± 1.25% and from 0.01 ± 0.01% to 0.12 ± 0.01%, respectively, though these changes were less pronounced than that of *Lactobacillus*.

At the species level, *E. faecium* and *L. rhamnosus* exhibited the most substantial decrease and increase in relative abundance, respectively, after fermentation using the probiotic complex (Fig. 3C). All *Enterococcus* species, including *E. faecium*, *E. durans*, and *E. gallinarum* had decreased relative abundances after fermentation. In contrast, most lactic acid bacteria species, including *Lacticaseibacillus rhamnosus*, *Lacticaseibacillus casei*, *Lactiplantibacillus plantarum*, and *Limosilactobacillus reuteri*, showed increased relative abundances after fermentation, while that of *Lactobacillus ruminis* decreased. Notably, *L. helveticus* was not detected in the gut dysbiosis-associated fecal microbiomes of either untreated or probiotic complex-fermented samples, despite *L. helveticus* IDCC 3801 being included in the probiotic complex. Instead, untreated samples harbored only *L. ruminis*, whose relative abundance dropped from 0.19 ± 0.02% to 0.03 ± 0.02% after fermentation. *Bifidobacterium animalis* and *B. breve* were absent from untreated fecal samples, whereas *B. longum* and *B. pseudocatenulatum* had relative abundance of 0.12 ± 0.02% and 0.05 ± 0.02%, respectively. After fermentation, *B. animalis*’ and *B. breve*’s relative abundance increased, whereas *B. longum*’s decreased to 0.05 ± 0.01% and *B. pseudocatenulatum*’s to 0.04 ± 0.04%. Additionally, *S. salivarius* was detected at 0.12 ± 0.01% after the fermentation treatment, whereas it was absent in the untreated fecal samples.

**Fig. 4:**
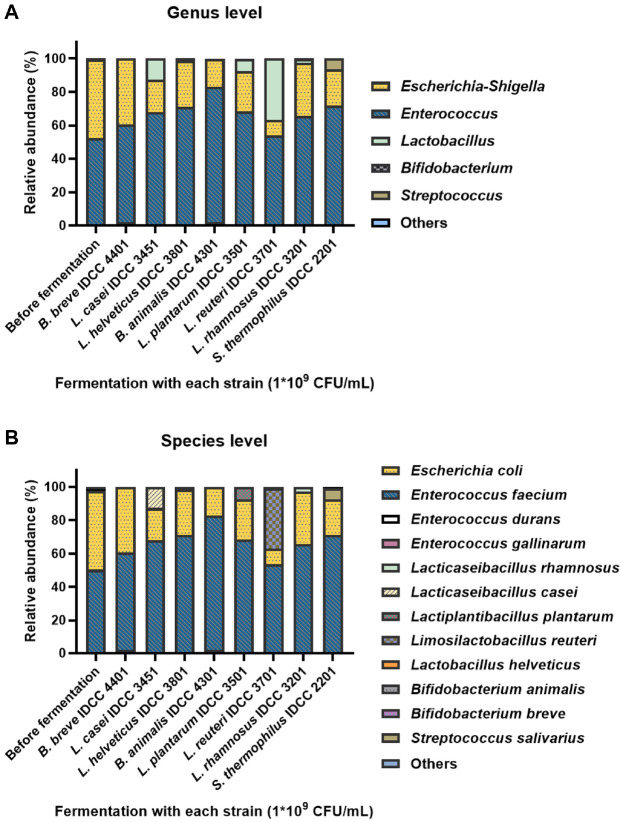
Taxonomic composition of single stran probiotic-fermented IBD fecal samples. Relative abundance (%) of microbial taxa at (**A**) genus and (**B**) species levels.

Interestingly, fermentation with the probiotic complex altered the genus- and species- level compositions of the gut dysbiosis-associated fecal microbiome to a much greater extent than the single-strain probiotic fermentations did (Fig. 4). None of the individual probiotic strains significantly reduced the relative abundances of *E. faecium* and *E. coli*. Moreover, *L. rhamnosus*, which had the highest relative abundance following probiotic complex fermentation, reached only 2.51% when fermented with *L. rhamnosus* IDCC 3201 alone. Similarly, *B. animalis* and *B. breve* exhibited minimal growth in single-strain fermentations with either *Bifidobacterium* species, despite their markedly higher abundances after probiotic complex fermentation, supporting putative synergistic or competitive interactions within the consortium.

**Fig. 5:**
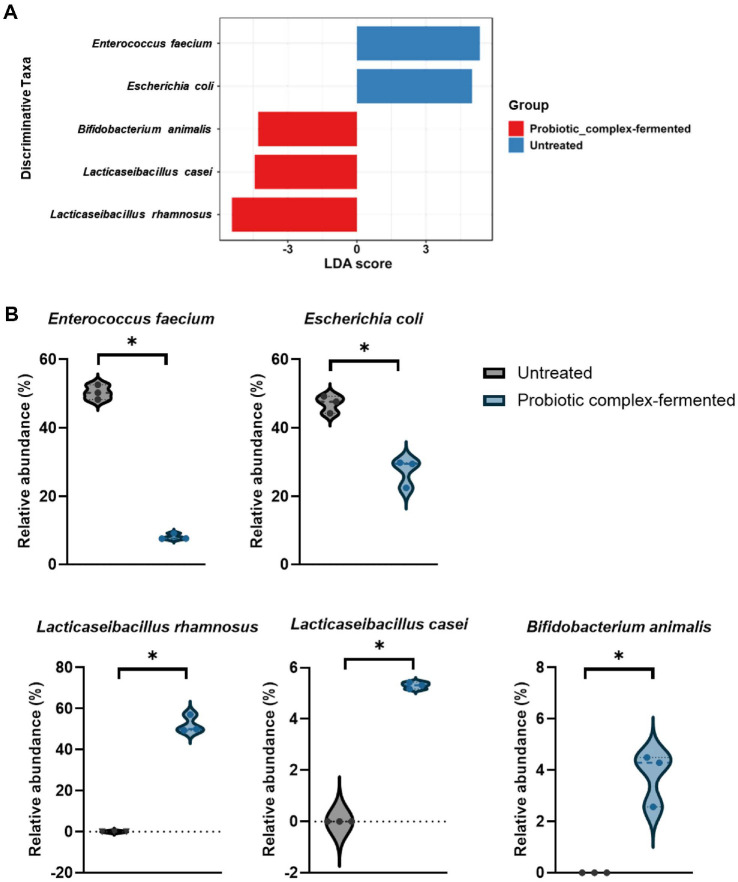
Enrichment analysis at the species level between untreated and probiotic complex-fermented IBD fecal samples using (**A**) linear discriminant effect size (LEfSe). (**B**) Species with significantly different relative abundances between groups were identified, with significant differences indicated as * (*p* < 0.05) using the Mann–Whitney U test.

LEfSe analysis identified five bacterial species as the most discriminative taxa contributing to the differences between the two groups. (Fig. 5A). These five species exhibited significantly different relative abundances between untreated and probiotic-complex fermented groups, suggesting their potential role as key microbial signatures associated with the intervention. *E. faecium* and *E. coli* were relatively more abundant in the untreated group, whereas *L. rhamnosus*, *L. casei*, and *B. animalis* were significantly enriched in the probiotic complex-fermented group, compared with its poor adaptation as a single strain, suggesting potential synergy or cross-feeding interactions, but does not confirm yet. These findings were further supported by violin plot analyses of the relative abundances (%) (Fig. 5B), which showed that the probiotic complex intervention significantly altered the microbial composition (*p* < 0.05, Mann–Whitney U test). Specifically, the relative abundance of *E. faecium* in untreated samples, which was 50.40 ± 2.16%, decreased to 8.14 ± 0.90% after the probiotic complex fermentation, and *E. coli*, which accounted for 47.08 ± 2.51% in the untreated samples, decreased to 27.31 ± 4.17% after the fermentation. While *L. rhamnosus*, *L. casei*, and *B. animalis* were not present in the untreated samples, they increased to 52.19 ± 4.32%, 5.31 ± 0.13%, and 3.78 ± 1.06%, respectively, by the fermentation of probiotic complex.

This observation was consistent with the results of the taxonomic composition analysis presented in Fig. 3. Therefore, these results provide additional evidence that may help interpret the lack of statistical significance observed in the diversity indices shown in Fig. 2.

### Metabolite Profiles of Gut Dysbiosis-Associated Fecal Samples after Fermentation with a Probiotic Complex

**Fig. 6:**
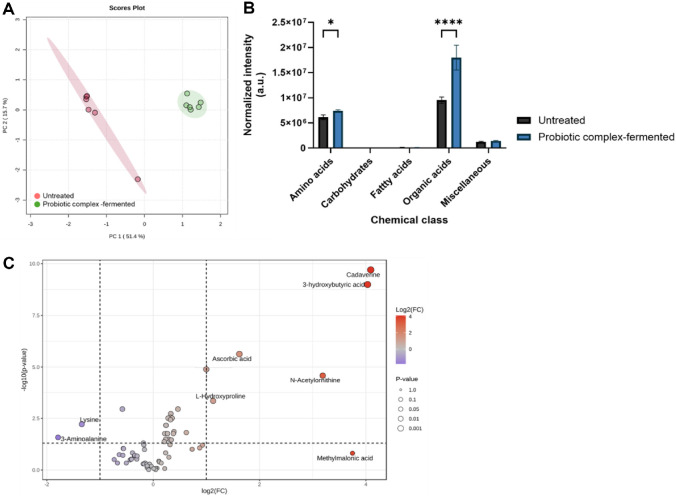
Overall metabolomic changes in untreated and probiotic complex-fermented IBD fecal samples, as assessed using (**A**) a principal component analysis, (**B**) the normalized intensities of the main chemical classes, and (**C**) a volcano plot. Normalized intensities of metabolites were compared between the untreated and probiotic complex–fermented groups within each chemical class. Statistical significance was assessed using a *t*-test, and significant differences are indicated by asterisks: *p* < 0.05 (*), *p* < 0.0001 (****).

To investigate metabolomic changes associated with probiotic complex fermentation, metabolites in the gut dysbiosis-associated fecal samples were analyzed (Fig. 6). The PCoA showed that the metabolite profiles in probiotic complex-fermented fecal samples differed significantly from those in untreated samples (Fig. 6A). PC1 accounts for the largest portion of variance, effectively separating untreated and probiotic-treated groups, whereas PC2 explains a smaller variance and does not distinctly separate the groups. The PC1 scores, in particular, differed significantly between untreated and fermented samples, with values of 1.25 ± 0.17 and −1.25 ± 0.53, respectively. Differences in PC scores indicate differences in total metabolite abundance between samples. Additionally, the PCoA model for probiotic complex-fermented samples had variation values (R^2^X) of 0.514 and 0.157, respectively, indicating a high level of discrimination between the samples. These results suggest that fermentation using the probiotic complex modulated metabolites in gut dysbiosis-associated patients’ feces.

A total of 75 fecal metabolites were measured and categorized into five chemical classes: amino acids, carbohydrates, fatty acids, organic acids, and miscellaneous (Fig. 6B). The relative abundance of each chemical class was calculated as the sum of the relative abundances of all metabolites belonging to that class. Probiotic complex-fermented fecal samples exhibited higher levels of metabolites associated with organic acids, amino acids, and carbohydrates than untreated samples. Six metabolites had log_2_ fold changes (FCs) > 1, with *p* < 0.05, while two metabolites had log_2_FCs < - 1 with *p* < 0.05 (Fig. 6C). Cadaverine exhibited the highest FC, increasing 17.04 ± 2.32 folds (*p* < 0.05), whereas 3-aminoalanine had the lowest FC at 0.29 ± 0.22 (*p* < 0.05). Thus, probiotic fermentation produced distinct metabolomic shifts, potentially reflecting altered microbial activities.

**Fig. 7:**
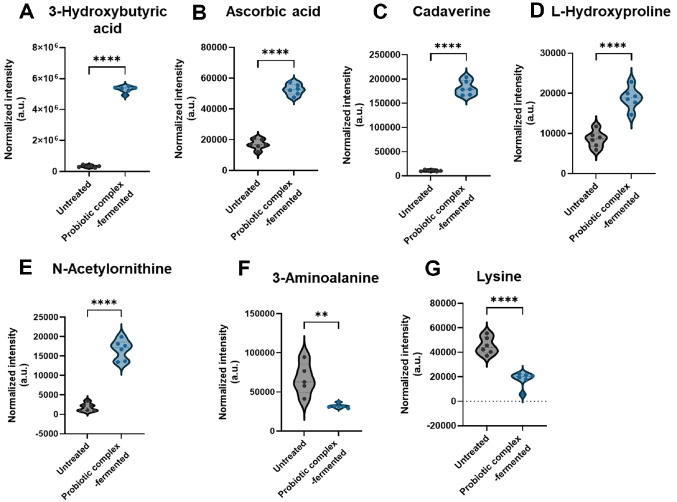
Key metabolites significantly altered by probiotic complex fermentation in IBD fecal samples. These metabolites were identified based on three criteria: (1) their presence in PubChem, (2) the statistical significance of the change due to fermentation (*p* < 0.05) (3) an (FC) threshold of log_2_FC > 1 or log_2_FC < - 1. Seven metabolites were included: (**A**) 3- hydroxybutyric acid, (**B**) ascorbic acid, (**C**) cadaverine, (**D**) L-hydroxyproline, (**E**) N-acetylornithine, (**F**) 3-aminoalanine, and (**G**) lysine. Statistical significance is indicated by asterisks: ** (*p* < 0.01) and **** (*p* < 0.0001).

The normalized intensity of each metabolite was also analyzed, and potential metabolites were distinguished from fecal components based on three criteria to identify metabolites that potentially contributed to improving the microbiomes of gut dysbiosis-associated fecal samples: (1) metabolites detected via gas chromatography-mass spectrometry were included in the PubChem database; (2) the statistical significance of the difference in the signal intensity of a given metabolite between untreated and probiotic complex-fermented samples should be at or above a 95% confidence level; and (3) the absolute value of the ratio of the metabolite’s normalized intensity in the fermented sample to its normalized intensity in the untreated sample should be greater than 2 (log_2_FC > 1 for upregulated metabolites) or less than 0.5 (log_2_FC < −1 for downregulated metabolites). According to these criteria, five upregulated and two downregulated metabolites were identified (Fig. 7). Specifically, the organic acids such as 3-hydroxybutyric acid, ascorbic acid, and cadaverine and the amino acids such as L-hydroxyproline and N-acetylornithine, exhibited increased normalized intensities after probiotic complex fermentation (Fig. 7A–7E), and in contrast, the amino acids including 3-aminoalanine and lysine decreased in intensity after probiotic complex fermentation (Fig. 7F–7G).

## Discussion

The gut microbiome plays a crucial role in gut dysbiosis. This study aimed to explore potential therapeutic strategies for gut microbial and metabolic imbalances through the administration of a probiotic complex. Specifically, we examined whether an eight-strain probiotic complex could modulate microbial composition and metabolic output in a dysbiosis-associated *in vitro* fermentation model derived from a single IBD donor.

The types and development of gut dysbiosis have been suggested to be influenced by SCFAs and their precursors, organic acids [25], as SCFAs in fecal samples vary by the gut dysbiosis-related symptoms [26]. These differences are associated with the distribution of gut microbiota, colonic absorption, and colon transit time [27]. Additionally, SCFAs regulate intestinal pH to inhibit the growth of pathogens and promote the growth of beneficial bacteria [28]. The immune system and intestinal inflammation are also influenced by SCFA concentration [29]. Our findings regarding SCFA production (Fig. 1) are consistent with previous studies showing that fecal samples from an adult with gut dysbiosis had significantly low levels of SCFA, but SCFAs [30, 31]. Acetate and propionate had an anti-inflammatory effect in cases of gut dysbiosis, such as IBD or Crohn’s disease [32]. Lactic acid and succinic acid are key organic acid precursors of propionic acid [33]. While most organic acids are metabolized into SCFAs that could contribute to gut health, lactic acid itself reduces colonization by pathogens and could act as a barrier to the growth of pathogenic bacteria [34], while succinic acid can improve glucose homeostasis through intestinal gluconeogenesis [35]. Therefore, the increases in organic acids and SCFAs after probiotic complex fermentation seen in the current study, likely due to the changes in the fecal microbiome caused by our eight-strain probiotic complex, demonstrate the complex’s potential to improve gut dysbiosis.

The observed increase in richness (Chao1 index) likely results from the introduction of the eight probiotic strains during fermentation. Conversely, the increase in Simpson’s index indicated reduced diversity and increased dominance of specific taxa, not a more balanced composition. While alpha and beta diversity analyses did not reveal statistically significant differences (*p* > 0.05), visual inspection of the PCoA plots and community composition suggested divergence between the two groups (Figs. 2 and 3). LEfSe analysis further supported this observation by identifying key taxa with significantly different relative abundances, suggesting potential biological relevance of group-specific microbial signatures (Fig. 5). Previous studies have also reported that microbial community structure showed remarkable group-specific patterns, despite a lack of statistical significance in alpha and beta diversity [36, 37]. In this study, the fecal microbial communities of gut dysbiosis-associated adult were dominated by the opportunistic bacteria *E. coli* and *E. faecium*, with low levels of beneficial bacteria, such as *Lactobacillus* and *Bifidobacterium* spp. Individual sample compositions are presented to illustrate inter-replicate variability. The predominance of *E. coli* and *E. faecium* is a characteristic of the *in vitro* fermentation environment and may not represent the *in vivo* dysbiotic microbiome. *Escherichia coli* is one of the most abundant species observed in gut dysbiosis-associated fecal microbiomes due to its high adhesive properties under inflammatory intestinal conditions [38, 39]. *Enterococcus faecium* has also been reported as one of the predominant species in the microbiome of gut dysbiosis-associated patients including IBD [40], and *E. faecium* infection may be associated with immune modulation by increasing pro-inflammatory cytokines [41]. Additionally, many gut dysbiosis-associated people such as IBD frequently take antibiotics to alleviate their symptoms, which may increase *E. faecium* abundance in their fecal microbiome due to its antibiotic resistance [42, 43]. Both *E. coli* and *E. faecium* are commensal bacteria under normal conditions, but they can act as opportunistic pathogens during intestinal dysbiosis [44-46]. *Lactobacillus ruminis*, the only *Lactobacillus* species detected in untreated fecal samples, is commonly found and identified in the microbiome associated with inflammation-driven dysbiosis [45, 47, 48]. Since *L. ruminis*, unlike other *Lactobacillus* species, contains pro-inflammatory flagellin proteins and adheres more strongly to the intestinal mucosa [49], it has been reported that *L. ruminis* can exhibit overgrowth and promote the inflammation in the intestinal tract when intestinal function is weakened [50]. Interestingly, metagenomic analysis revealed that *L. helveticus* IDCC 3801, a constituent strain in the probiotic complex, was not present in fermented fecal samples. Its abundance might have remained below the detection threshold of the metagenomic method, particularly if its growth was limited during fermentation. Although direct evidence is limited, some studies suggest that *L. helveticus* might exhibit lower tolerance to stress condition, making it less competitive in complex microbial environments [51]. This could result in competitive exclusion by more dominant native or probiotic *Lactobacillus* strains during fecal fermentation. It should be noted that the high relative abundance of *Lactobacillus* species observed in the probiotic-treated group may be exaggerated due to the simplified nature of the *in vitro* system, which lacks host physiological constraints. Further strain-specific quantitative polymerase chain reaction (qPCR) or culture-based validation would be needed to clarify this growth limitation.

After fermentation with the eight-strain probiotic complex, the gut dysbiosis-associated fecal microbiome composition became more balanced than after single-strain probiotic fermentation. Although few studies have investigated the ability of probiotic complexes to improve inflammatory intestinal conditions at the *in vitro* level, multi-species probiotics have shown higher efficacy as a gut dysbiosis treatment than single-strain probiotics [52-54].

Fecal sample from an IBD patient exhibited metagenomic changes and metabolome changes [55, 56]. In our results, 3-hydroxybutyric acid, ascorbic acid, cadaverine, L-hydroxyproline, and N-acetylornithine increased, while 3-aminoalanine and lysine decreased in the gut dysbiosis-associated fecal samples after probiotic complex fermentation. A representative metabolite of fatty acid oxidation, 3-hydroxybutyric acid could serve as a substrate for polyhydroxybutyrate synthesis in bacteria and has been identified as a key metabolite for the prevention of colonic inflammation, including that of IBD [57-59]. In addition, it was previously reported that probiotic supplementation helped improve intestinal inflammation by increasing serum 3-hydroxybutyrate [60]. Ascorbic acid, also known as vitamin C, is reduced during gut inflammation and must be supplied exogenously because the human body does not possess the gene encoding gluconolactone oxidase, which synthesizes vitamin C [61-63]. A high abundance of *E. coli* in the fecal microbiome could contribute to dysregulating the expression of intestinal transporters, such as the microRNA regulators of SVCT1 and SVCT2, inhibiting ascorbic acid metabolism in the gut dysbiosis [64]. Therefore, in the current study, probiotic complex fermentation may have reduced the abundance of *E. coli* and increased ascorbic acid levels in the gut dysbiosis-associated fecal microbiome, and ascorbic acid may have, in turn, enhanced the abundance of *Bifidobacterium* spp. [65]. Cadaverine is a biogenic polyamine produced from lysine-by-lysine decarboxylase, and it has been reported that increased cadaverine synthesis might protect the human body under stress conditions [66]. However, cadaverine levels are known to be elevated in inflammatory conditions such as ulcerative colitis, Crohn’s disease, and IBD [67]. In our study, untreated fecal samples exhibited high lysine and low cadaverine levels, whereas probiotic treatment appeared to restore lysine decarboxylation activity, leading to increased cadaverine and decreased lysine. Further mechanistic studies are needed to elucidate these context-dependent roles. The amino acid L-hydroxyproline plays a critical role in intestinal barrier integrity and intestinal function through proline metabolism [68], and it is known to have various effects on the composition of the gut microbiome and its interactions with the host [69, 70]. Specifically, in DSS-induced colitis rat models, an imbalance in proline metabolism, resulting in intestinal barrier dysfunction [71]. N-acetylornithine is an intermediate product of the arginine and proline metabolism, and its levels were significantly changed in the case of gut dysbiosis [72, 73]. In a previous study, treating IBS-C patients with *L. rhamnosus* IDCC 3201 increased levels of N-acetylornithine, which was identified as a key fecal metabolite for improving gut dysbiosis [74]. Although no research illuminated the role of 3-aminoalanine in the gut, alanine has potential as a co-agonist of N-methyl-d-aspartate receptors (NMDARs), which play a crucial role in the nervous and endocrine systems [75], suggesting that it might be important in gut dysbiosis via the gut-brain axis [76]. Therefore, probiotic complex fermentation may help improve the altered metabolome of fecal samples associated with the abnormal intestinal conditions. Although our study lacks a healthy control group, we interpreted observed changes—such as increased levels of beneficial taxa (*e.g.*, *Bifidobacterium* spp. and *Lactobacillus* spp.) and enhanced production of SCFAs and other beneficial metabolites—as indicative of a shift toward a more balanced gut environment. According to Van Hul *et al*., a healthy gut microbiome is typically characterized by high microbial diversity, the presence of beneficial microbial taxa, functional resilience, and the production of health-promoting metabolites such as SCFAs and bile acid derivatives. These characteristics are central to gut health and are associated with immune regulation and intestinal barrier integrity. While our *in vitro* model lacks direct comparison with healthy donor samples, our findings align with key hallmarks of a healthy gut ecosystem. Future studies incorporating healthy reference groups and *in vivo* validation are necessary to confirm these preliminary observations [77].

In this study, we analyzed the treatment effects of a multi-species probiotic complex, including *Lactobacillus* spp., *Bifidobacterium* spp. and *Streptococcus* spp., on the microbiome and metabolome of gut dysbiosis-associated fecal samples. Unlike previous studies that primarily focused on clinical effects, this study provided a more detailed understanding of the gut dysbiosis and elucidated the metagenomic and metabolic effects of probiotic complex. However, the ability of the probiotic complex to alleviate gut dysbiosis-associated symptoms needs to be confirmed through human clinical trials. Further studies should demonstrate the therapeutic efficacy of the probiotic complex formulations for gut dysbiosis-associated adult with respect to host-microbe signaling, the host immune response, and the complex physiological environment of the human gastrointestinal tract. Also, the specific mechanism of the interactions between the probiotic strains should be revealed to prove the synergistic effects of the probiotic complex. In this study, a 24-h fermentation time point was selected based on previous *in vitro* models simulating colonic conditions [78]. While this approach provides a standardized and practical framework for evaluating microbial and metabolic outcomes, we acknowledge that fermentation is a dynamic process, and additional time points may offer further insights into temporal changes in microbial and metabolite profiles. Moreover, this study was conducted using technical replicates derived from a single gut dysbiosis-associated donor sample. Therefore, the results should be interpreted as preliminary, and further validation using biological replicates from multiple donors is necessary to confirm the robustness and generalizability of these findings.

## Conclusion

The fermentation of feces from an imbalanced fecal microbiota using a multi-species probiotic complex enhanced the proliferation of beneficial bacteria while suppressing opportunistic pathogens. Additionally, it modulated the metabolism of organic acids and amino acids by improving the overall composition of the gut microbiome. These findings suggest that fermentation using a probiotic complex may contribute to restoring microbial balance, potentially alleviating symptoms associated with gut dysbiosis. These descriptive findings support further multi-donor and *in vivo* studies to test whether probiotic consortia can reproducibly modulate dysbiotic ecosystems and ameliorate gut dysfuctions. Also, the specific mechanism of the interactions between the probiotic strains should be revealed to prove the synergistic effects of the probiotic complex.
